# Primary Congenital Lymphedema with More Than 10 Years of Treatment Using the Godoy Method Through to Adolescence

**DOI:** 10.3390/pediatric13010012

**Published:** 2021-02-20

**Authors:** Ana Carolina Pereira de Godoy, Jose Maria Pereira de Godoy, Maria de Fatima Guerreiro Godoy

**Affiliations:** MD Resident of the Pediatrics Unit Intensive Therapy of Santa Casa de São Paulo, Brazil and Research Group of Clínica Godoy, São Jose do Rio Preto 15025120, Brazil; acp.godoy@gmail.com (A.C.P.d.G.); mfggodoy@gmail.com (M.d.F.G.G.)

**Keywords:** lymphedema, treatment, children, monotherapy, congenital

## Abstract

Introduction: Lymphedema is a specific type of edema that affects regions of the body in a chronic, progressive manner. Aim: The aim of the present study was to describe the therapeutic evolution of more than ten years of treatment for primary congenital lymphedema using the Godoy and Godoy method. Method: Ten children with primary congenital lymphedema with more than ten years of treatment at the Godoy Clinic were evaluated. Children with a clinical diagnosis of primary congenital lymphedema in treatment for more than 10 years with the Godoy Method. Cervical stimulation is the first treatment option of the method and is performed as monotherapy. The patients were reevaluated with weekly, bi-weekly and monthly frequencies and then every three months or when the family was able to return to the clinic. Results: For cases in which cervical stimulation was not possible, grosgrain stockings as monotherapy was the second therapeutic option. Conclusion: The Godoy and Godoy Method is effective at reducing edema in cases of primary congenital lymphedema, with the maintenance of the results throughout the treatment period.

## 1. Introduction

Lymphedema is a specific type of edema that affects regions of the body in a chronic, progressive manner [1]. The cause may be congenital (denominated primary) or secondary. There are few studies in the literature on the epidemiology of primary lymphedema, but this is a rare condition that affects approximately 1.5 out of every 100,000 children in the USA, with puberty associated with the emergence of early onset edema [2,3]. A population-based study detected chronic edema in 1.33 of every 1000 individuals, increasing to 5.4 for every 100 patients over 65 years of age [4,5]. The classification of congenital lymphedema is based on age: primary congenital lymphedema emerges prior to two years of age; early primary lymphedema emerges between two and 35 years of age; and late onset congenital lymphedema emerges after 35 years of age.

There was previously no specific treatment option for primary congenital lymphedema, but Godoy and Godoy developed a novel concept of lymphatic stimulation in recent years, denominated cervical lymphatic therapy or the Godoy cervical stimulation method. As a monotherapy, this treatment option enables the normalization or near normalization of edema in children [5,6,7,8].

Stockings made from grosgrain fabric constitute another advance in the treatment of lymphedema, enabling normalization or near normalization as a monotherapy. Several studies have proven the effectiveness of these stockings with regard to the reduction in edema and the maintenance of the results. Another study has proven the effectiveness of these stockings when adapted for use on children to reduce edema [9,10].

Manual lymphatic therapy is a novel concept in lymphatic drainage based on linear movements and therefore following scientific concepts of the drainage of a linear collector [11]. Mechanical lymphatic therapy with the RAGodoy^®^ device (São Paulo, Brazil) is another lymphatic drainage concept based on plantar flexion and extension. Several studies have demonstrated the effectiveness of this device in the reduction of edema as well as the formation and drainage of lymph [12].

The Godoy and Godoy method for the treatment of primary congenital lymphedema enables the normalization or near normalization of edema. The first option is monotherapy using cervical lymphatic therapy. Grosgrain stockings adjusted and adapted to each child throughout the entire therapeutic process as monotherapy is the second option. Cervical lymphatic therapy combined with grosgrain stockings is another option and mechanical lymphatic therapy with the RAGodoy^®^ device is the fourth option (São Paulo, Brazil).

The aim of the present study was to describe the therapeutic evolution of more than ten years of treatment for primary congenital lymphedema using the Godoy and Godoy method.

## 2. Methods

### Patients

Ten children with primary congenital lymphedema with more than ten years of treatment at the Godoy Clinic-Sao Jose do Rio Preto-Brazil were evaluated.

## 3. Inclusion Criteria

Children with a clinical diagnosis of primary congenital lymphedema in treatment for more than 10 years with the Godoy Method.

## 4. Exclusion Criteria

Children with secondary lymphedema and those who had not been in treatment for ten years.

## 5. Development

The diagnosis of primary congenital lymphedema was clinical and based on foot circumference. Edema on the dorsum of the foot and positive Steimmer’s sign were the main clinical signs. In cases of doubt, the children were followed up clinically and those for whom confirmation was achieved were included in the treatment.

Cervical stimulation is the first treatment option of the method and is performed as monotherapy. The parents were trained under the supervision of a professional and those who proved capable were free to perform the method every day for 15 min. The patients were reevaluated with weekly, bi-weekly and monthly frequencies and then every three months or when the family was able to return to the clinic. For cases in which cervical stimulation was not possible, grosgrain stockings as monotherapy was the second therapeutic option. These stockings are hand-crafted and custom-made for each child. The parents were trained on how to place the stocking and make the necessary adjustments with the reduction of the edema. The stockings were changed when they become too small due to the motor development and growth of the child. For some children, both cervical stimulation and grosgrain stockings were used. Others had the option of mechanical lymphatic therapy with the RAGodoy^®^ device when they became older. This device performs passive plantar flexion and extension movements. The study was approving for Ethical Committee Research of Medicine School of Sao Jose do Rio Preto # CAAE 03001318.0.0000.5415.

## 6. Results

The sample was composed of four males and two females with a mean age of 16.3 years. The patients had been followed up for 13.2 years. Four underwent cervical stimulation alone. Two used both cervical stimulation and grosgrain stockings and four underwent a combination of cervical stimulation and mechanical lymphatic drainage (RAGodoy^®^ device, São Paulo, Brazil).

## 7. Discussion

The present study reports the therapeutic evolution of more than 10 years of follow up using the Godoy Method for the treatment of primary congenital lymphedema. Children with early congenital and secondary lymphedema were excluded from the study. While the literature offers no specific treatment for these children, Godoy and Godoy propose cervical lymphatic therapy and the use of handcrafted grosgrain stockings either as monotherapy or combined. Studies have shown that cervical lymphatic therapy as monotherapy enables the normalization or near normalization of edema in two years of treatment. When combined with grosgrain stockings, a synergic effect is found on the reduction in edema [6].

What we have observed throughout these ten years is that all children currently have limbs within or near the range of normality. However, only four children continued using cervical lymphatic stimulation as monotherapy. The combination of this treatment option and the RAGodoy^®^ device was at the request of the parents, who wanted another form of treatment for their children. The combination with grosgrain stockings was another option for some patients. However, we have found that cervical stimulation alone enables normalization or near normalization in nearly all cases. The stockings seem to be an option in cases of edema in a more advanced stage and enable reducing the treatment time.

Over the years, we have found that some children have been without cervical lymphatic therapy for more than five years and continue to be within the range of normality. This suggests the possibility of a cure for lymphedema, but the follow up of a greater number of children for a longer period of time is required to confirm this hypothesis. Nonetheless, such findings have led us to prioritize cervical lymphatic stimulation as monotherapy.

The combination of therapies initially occurred with the aim of accelerating the reduction of the edema, which actually does happen. However, many parents end up abandoning cervical stimulation and only use the grosgrain stockings. This is not a good decision, because the results of cervical stimulation seem to be the best option.

The combination with the RAGodoy^®^ device is another option that contributes to the reduction in edema and maintenance of the results. However, we do not suspend cervical lymphatic therapy in such cases. When the three treatment options are combined, the reduction is even faster. The intensive form of treatment for primary congenital lymphedema is another option and enables reaching normalization or near normalization in a few days. However, the difficulty of lodging the children for long periods is a limiting factor. This study shows that the Godoy Method is a therapeutic option that enables the normalization or near normalization of the limbs with edema and the maintenance of the results.

It is also important to consider the psychological aspect. Cervical lymphatic therapy alone does not require an additional therapy. In contrast, the stockings can cause embarrassment for the children. Mothers are counseled to take care in terms of infection. Whenever a child is at risk, such as the presence of mycosis or a skin lesion, the parents should seek medical attention as soon as possible. The child is orientated to have a life as close as possible to normal. Physical activities, such as exercise, are permitted, but should begin under the supervision of the treatment team.

One of the patients was an adolescent who had had approximately 200 mL of edema in the upper limbs, which was reduced with treatment of the lower limb lymphedema during three weeks of intensive treatment. The patient has maintained the results for approximately six years using grosgrain stockings and the RAGodoy device, with no specific therapy for the upper limbs. This suggests that treatment in one extremity of the body can affect the entire body [8].

## 8. Conclusions

The Godoy and Godoy Method is effective at reducing edema in cases of primary congenital lymphedema, with the maintenance of the results throughout the treatment period.

## Data Availability

This research does not apply.
